# Diosgenin and 4-Hydroxyisoleucine from Fenugreek Are Regulators of Genes Involved in Lipid Metabolism in The Human Colorectal Cancer Cell Line SW480

**DOI:** 10.22074/cellj.2021.6751

**Published:** 2020-04-22

**Authors:** Maryam Mohammad-Sadeghipour, Mehdi Mahmoodi, Mojgan Noroozi Karimabad, Mohammad Reza Mirzaei, Mohammad Reza Hajizadeh

**Affiliations:** 1. Student Research Committee, School of Medicine, Kerman University of Medical Sciences, Kerman, Iran; 2. Department of Clinical Biochemistry, Afzalipoor Faculty of Medicine, Kerman University of Medical Sciences, Kerman, Iran; 3. Department of Clinical Biochemistry, Faculty of Medicine, Rafsanjan University of Medical Sciences, Rafsanjan, Iran; 4. Molecular Medicine Research Center, Institute of Basic Medical Sciences Research, Rafsanjan University of Medical Sciences, Rafsanjan, Iran

**Keywords:** Trigonella, Diosgenin, Orlistat, Obesity

## Abstract

**Objective:**

Diosignin and 4-hydroxy-L-isulosine (4-OH-Ile) are the two active ingredients of Fenugreek (*Trigonella foenum- graecum*). Thus, in this study, we examined the effects of hydroalcoholic extract of fenugreek seeds (HEFS), diosgenin and 4-OH-Ile on the expression of acetyl-CoA carboxylase (*ACC*), fatty acid synthase (*FAS*), peroxisome proliferator-activated receptor gamma (*PPARγ*) and low-density lipoprotein (LDL) receptor (*LDLR*) which are involved in lipid metabolism in SW480 cell line.

**Materials and Methods:**

In this experimental study, SW480 cells were cultured in RPMI-1640 medium and treated with HEFS, diosignin, 4-OH-Ile or orlistat for 24 and 48 hours. Inhibitory concentration of 20% (IC20) was calculated using MTT method and cells were then pre-treated with the IC20 concentrations for 24 and 48 hours before RNA extraction and cDNA synthesis. Changes in the expression of *ACC, FAS, PPARγ* and *LDLR* genes were assayed by employing the real time-polymerase chain reaction (PCR) method.

**Results:**

Our results showed a significant down-regulation in the expression of ACC (P<0.001 and P<0.001 after 24 and 48 hours, respectively) and *FAS* genes (P<0.001 and P<0.001 after 24 and 48 hours, respectively) in SW480 cells treated with HEFS, diosignin, 4-OH-Ile, or orlistat, but significant up-regulation in the expression of *PPARγ* (P<0.001 and P<0.001 after 24 and 48 hours, respectively) and *LDLR* (P=0.005 and P=0.001 after 24 and 48 hours, respectively).

**Conclusion:**

According to the results of the present study, HEFS, diosgenin and 4-OH-Ile up or down-regulate the expression of some predominant genes involved in lipid metabolism pathway, similar to that observed for orlistat. These types of regulatory effects are presumably proper for the treatment of obesity and overweight.

## Introduction

**Fig.1:**
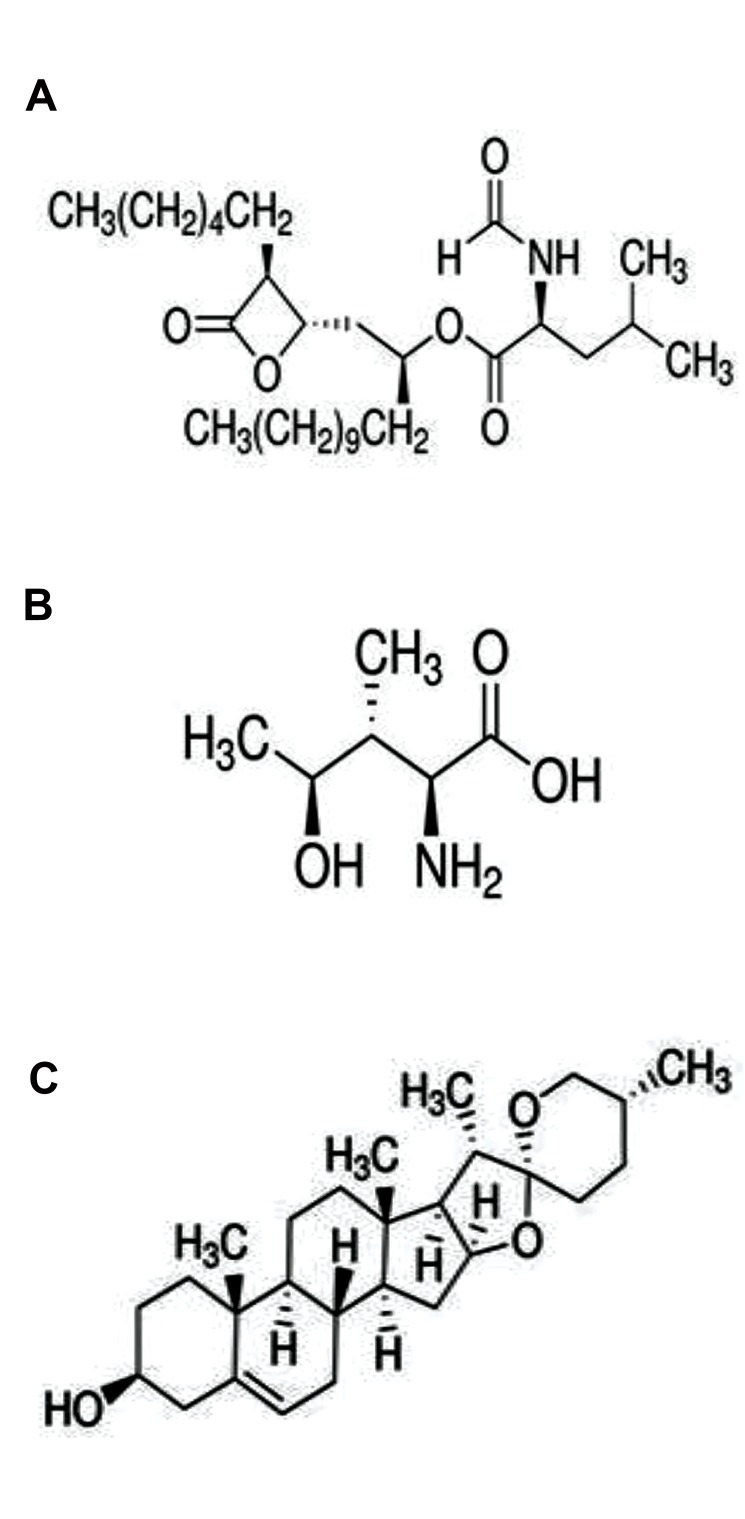
The structure of antihyperlipidemic drug and bioactive compounds of fenugreek respectively. **A.** Orlistat, **B.** 4-hydroxy-L-isoleucine, and **C.** Diosgenin.

Obesity is one of the greatest public health challenges of the 21^st^ century that is increasing at various rates worldwide (1). Approximately 20% of the global population is obese (about 1.5 billion people) (2). Obesity has an adverse effect on the quality of life and overweight is associated with some disorders such as dyslipidemia, type 2 diabetes mellitus, hypertension, gallbladder disease (3), osteoarthritis and cancers at several sites (mainly endometrial, breast, and colorectal) (4). Overweight is defined as having a body mass index (BMI) between 25 and 29.9 kg/m^2^, while obesity is described as a BMI of over 30 kg/m^2^ (5). Four weight-loss drugs have recently been approved by the US food and drug administration (FDA), and among them, some drugs (Orlistat, Xenical® and Alli® and Sibutramine) were found to be appropriate for longterm use (6). Orlistat as a reversible inhibitor of gastric and pancreatic carboxylester lipase also reduces the absorption of lipids in the intestine (Fig.1A) (7). In addition to being expensive, these synthetic reagents have considerable side effects on the gastrointestinal tract and their use is restricted to treatment of obesity. Medicinal plants are of great value and importance and are considered for providing health and well-being, both for treatment and prevention of the diseases and therefore, many of the drugs of modern medicine were originated from plant sources (8).

*Trigonella foenum-graecum* (Fenugreek) is an annual plant belonging to the Leguminosae family, and grows in different climates especially in the Mediterranean countries and India. The fenugreek seeds have long been consumed for medicinal purposes in many countries (9). The biological and pharmaceutical properties of fenugreek seeds are mainly due to the presence of several components, including alkaloids, sapogenins, mucilages, 4-hydroxy-L-isoleucine (4-OH-Ile) (Fig.1B), galactomannan, diosgenin (Fig.1C) and fiber (10). Findings of a study showed that daily consumption of 1176 mg of fenugreek hydroalcoholic extract by healthy volunteers, resulted in decreased fat intake (9). Fuller and Stephens (11), reported that three bioactive compounds of fenugreek (diosgenin, 4-OH-Ile and fiber) controlled both hyperglycemia and hyperlipidemia. Fenugreek is also a rich source of diosgenin (as a steroidal saponin) which is generated by hydrolysis of saponins (12). 4-OH-Ile is a branched-chain amino acid that exists in plant sources and is especially abundant in fenugreek seeds. Animal studies demonstrated hypoglycaemic and antihyperlipidemic properties for 4-OH-Ile (13) .

The absorption of fat by the gut cells has an important role in the maintenance of fat metabolism balance, but its regulation at the molecular level remains largely unknown (14). The gut cells play a role in the production of apolipoproteins and lipoproteins, which are formed of combination of lipids with proteins (15). Different proteins affect the absorption of fat in the gut, including: FAS, a key enzyme in fat biosynthesis (16), and *ACC* which is the key enzyme in fat metabolism, it is a biotin-dependent enzyme that catalyzes the irreversible carboxylation of acetyl- CoA to produce through its two catalytic activities, biotin carboxylase (BC) and carboxyltransferase (CT) (17). So, these cells are highly involved in the synthesis and absorption of fat. Moreover, orlistat, a gastric and pancreatic lipase inhibitor that reduces dietary fat absorption, has been used for nearly ten years (7), and is known as a FAS inhibitor (18). Since our aim was to study the hypolipidemic effects of HEFS and diosignin and 4-OH-Ile compared to orlistat, we preferred to use SW480 cell line.

Although metabolic effects of fenugreek have been widely studied, there is no study yet to address its effects on the gut cells. While most studies done in the SW480 cell line are related to cancer and metastasis and inflammation, there is no study of lipid metabolism in these cells yet. Thus, the current investigation was aimed for the first time, to examine the hypolipidemic effects of hydroalcoholic extract of fenugreek seeds (HEFS) and its two bioactive compounds (diosignin and 4-OH-Ile), in addition to orlistat via evaluation of the expression of acetyl-CoA carboxylase (ACC), fatty acid synthase (FAS), peroxisome proliferator-activated receptor gamma (PPARγ) and low-density lipoprotein (LDL) receptor (LDLR) as the genes responsible for lipid metabolism in the SW480 cell line.

## Materials and Methods

In this experimental study, a batch of SW480 cell line was purchased from Pasteur Institute (Iran, Tehran). RPMI- 1640, fetal bovine serum (FBS), penicillin-streptomycin and trypsin were provided from Gibco-BRL (Grand Island, NY, USA), MTT powder and dimethyl sulfoxide (DMSO) were bought from Merck (USA), diosgenin of 93% purity, 4-OH-Ile (2S3R4S Isoform) of ≥98% (TLC) purity and orlistat of approximately ≥98% purity, were provided from Sigma (USA). RNA extraction and cDNA synthesis kits were purchased from PARS Tous (Iran), and SYBR Green Premix Ex Taq II Kit was obtained from ABI Company (Takara, Japan). This study approved by the Rafsanjan University of Medical Sciences (RUMS) Ethical Committee (IR.RUMS.REC.1395.109).

### Extraction of plant materials

The dry milled fenugreek powder (5 g) was packed in a filter paper and placed into a container filled up to two thirds of its volume with 70% ethanol. The extraction was further performed using a Soxhlet apparatus at 80°C for 100 min (BAKHSHI Laboratory Industrial Co., Iran). The extract was dehydrated by a freeze dryer apparatus (VaCo5-D, Zirbus Technology Co., Germany) at -70°C for 72 hours and the collected dry yellow crystalline powder was stored at -20°C for further use. Different concentrations of the extract were obtained by dissolution in RPMI 1640.

### Preparation of diosgenin, 4-OH-Ile, and orlistat

Initial stock solutions of diosgenin, 4-OH-Ile, and orlistat were prepared in ethanol, phosphate-buffered saline (PBS), and DMSO, respectively.

### Cell culture

SW480 cells were cultured at 37°C in the presence of 95% O_2_ and 5% CO_2_ in complete cell culture medium (CCM) comprising RPMI-1640, in addition to 10% FBS and 100 IU/ml of penicillin and 100 μg/ml of streptomycin. Cells were grown to 80% confluence prior to treatment for 24 and 48 hours.

### Analysis of cell proliferation by MTT assay

Cell proliferation was assessed by 3-(4,5-dimethylthiazol- 2-yl)-2,5-diphenyltetrazolium bromide (MTT) assay. SW480 cells (7×10^3^ cells per well) were seeded in 96-well plates with the culture medium containing FBS, allowed to grow and become attached, and then treated with HEFS (0-2000 μg/ml), diosgenin (0-32 μg/ml) 4-OH-Ile (0-16 μg/ml) and orlistat (0-48 μg/ml) for 24 and 48 hours. All the experiments were performed in sextuple assay. After incubation, 10 μl of MTT solution (5 mg/ml in PBS stock solution) was added to each well and incubated at 37°C for 4 hours. The medium was removed, and the purple formazan crystals were dissolved in 150 μl of DMSO. The optical density (OD) was measured at 570 nm using an ELISA reader.

Relative growth rate (%)=(OD treatment/OD control)×100

An average inhibitory concentration of 20% (IC20) will result in 80% cell survival. The IC20 value was partly non-toxic where the SW480 cells exhibited an approximate viability of 80%. This IC20 concentration was considered for future treatment of SW480 cells. Thus, treatments were performed using HEFS 50 μg/ ml, and 6.21, 1.37, 4.64 μg/ml of diosgenin, 4-OH-Ile, and orlistat, respectively for 24 and 48 hours. One flask containing cells and complementary culture medium were considered as controls.

### RNA extraction and cDNA synthesis

Total cellular RNA content was isolated and the complementary DNA (cDNA) was synthesized employing Pars Tous kit according to the manufacturer’s instructions. Both purity and integrity of harvested RNA specimens were analyzed by spectrophotometry and electrophoresis in agarose gel, respectively. The purity was assessed by the A260/280 and A260/230 absorbance ratios obtained using a NanoDrop spectrophotometer. The samples were further used for cDNA synthesis.

### Real time-polymerase chain reaction

Specific primers were designed employing primer 3 and BLAST software in NCBI (Table 1). The level (percentage) of changes in the expression of *ACC*, *FAS*, *PPARγ*, and *LDLR* genes was evaluated by real time-polymerase chain reaction (RT-PCR) technique with ABI Step One Plus TM Real-Time PCR System (Applied Biosystems, USA) and using the Takara Bio SYBR Green Master Mix Kit (Japan) at a final volume of 20 μl. Thermal cycling conditions were as follows: 95˚C for 30 seconds and 40 cycles at 95˚C for 5 seconds, and continued at *ACC*: 60˚C, *FAS*: 62˚C, *PPARγ*: 58˚C, and *LDLR*: 61˚C for 30-60 seconds. Threshold cycle (CT) data was analyzed by Step One ver.2.3 software. Relative values of the fold changes in the expression of genes were calculated by 2^-∆∆Ct^ where ∆_Ct_=_Ct_ (target genes) - Ct (reference gene) and ∆∆_Ct_=∆_Ct_ (treated groups) - ∆_Ct_ (untreated group (control)). Eventually, 2^-∆∆CT^ values were estimated using Excel 2013 (Table 2) (19).

**Table 1:** Nucleotide sequence of primers used in this study


Gene	Primer sequence (5´-3´)

*ACC*	F: GGATCCGGCGCCTTACTT
	R: CTCCGATCCACCTCATAGTTGAC
*FAS*	F: TTGGAAGGCCTGCATCATG
	R: CACCTGGAGGACAGGGCTTA
PPARγ	F: TCAGGGCTGCCAGTTTCG
	R: GCTTTTGGCATACTCTGTGATCTC
*LDLR*	F: ACTGGGTTGACTCCAAACTTCAC
	R: GGTTGCCCCCGTTGACA	
*β-Actin*	F: GATCAGCAAGCAGGAGTATG
	R: GTGTAACGCAACTAAGTCATAG


**Table 2:** Inhibitory concentration of 20% (IC20) following 24 and 48 hours of treatment with HEFS, diosgenin, 4-OH-Ile, and orlistat


Treatment	IC20 after treatment (24, 48 hours)

HEFS (µg/ml)	50
Diosgenin (µg/ml)	6.21
4-OH-Ile (µg/ml)	1.37
Orlistat (µg/ml)	4.64


### Statistical analysis

Data is presented as mean ± SD of triplex independent experiments. Data were statistically analyzed by the SPSS Statistical Package software version 18.0 for Windows (SPSS Inc. Chicago, IL, USA). The gene expression data was analyzed by one-way ANOVA among different groups. Tukey’s post hoc test was used to evaluate differences in each group. Treated groups were compared to the untreated control using one-way ANOVA accompanied by a Dunnett’s post hoc test. Independent t test was used to compare the effect of treatment period in each group. The differences were considered significant if P<0.05.

## Results

### Effects of HEFS, diosgenin, 4-OH-Ile and orlistat on the viability of SW480 cells

**Fig.2:**
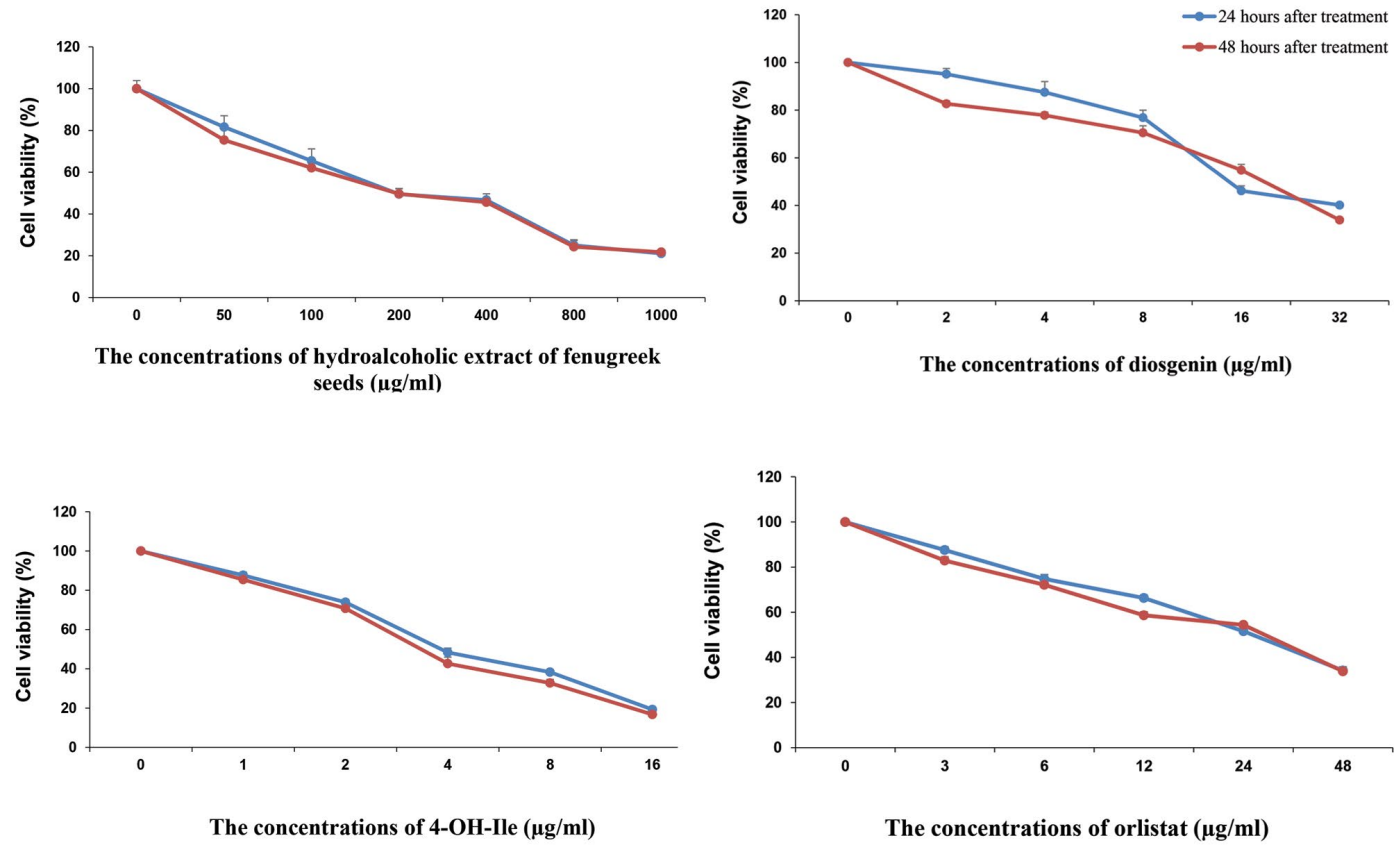
Percentage of SW480 cells viability following 24 and 48 hours of treatment with different concentrations of HEFS, Diosgenin, 4-OH-Ile, and Orlistat (μg/mL) measured by MTT assay. Results were obtained from three independent experiments as individual and triplicate and data are presented as mean ± SD, (P<0.001).

The *in vitro* cytotoxic effects of HEFS, diosgenin, 4-OH-Ile, and orlistat were evaluated by MTT test. Cell viability following treatment with different concentrations of the mentioned compounds, was assessed by MTT assay and is presented in Figure 2. These results showed that in response to 24 and 48 hours treatment with the mentioned compounds, the viability of SW480 cells was decreased in a concentration-dependent manner (P<0.001). Also, 24 hours after the treatment with HFSE, cell viability percentage decreased from 81.61 ± 5.44% at the concentration of 50 μg/mLl to 21.11 ± 1.40% at the concentration of 1000 μg/ml, and 48 hours after the treatment, it decreased from 75.38 ± 3.88% at the concentration of 50 μg/ml to 21.77 ± 2.96% at the concentration of 1000 μg/ml (P<0.001, Fig.2).

**Fig.3:**
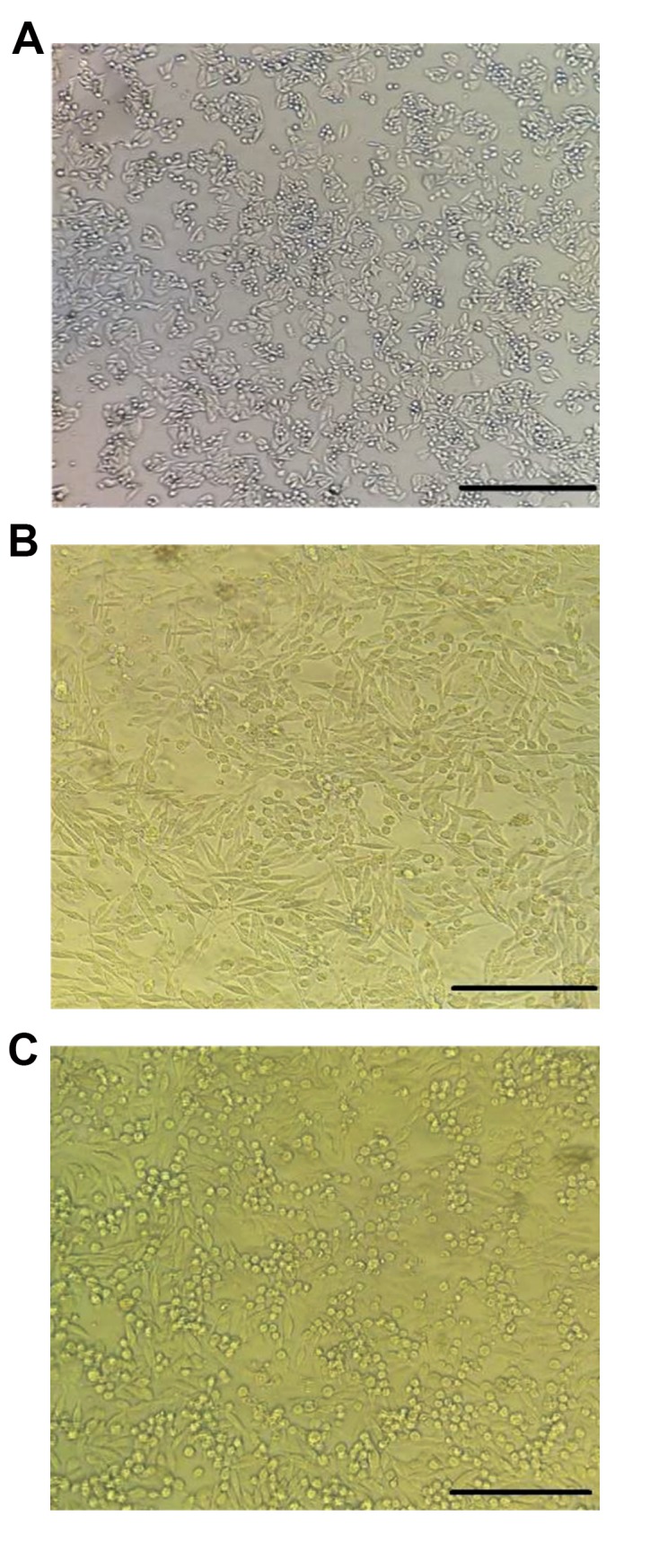
Morphological changes on SW480 cells after exposure with HEFS that were observed with an inverted microscope. **A.** 0 (untreated), **B.** IC20 concentration, and **C.** IC50 concentration (scale bar: A-C: 40 μm). IC; Inhibitory concentration.

The results also showed that 24 hours after the treatment with diosgenin, viability percentage decreased from 95.8 ± 2.35% at the concentration of 2 μg/ml to 40.16 ± 2.08% at the concentration of 32 μg/ml, and 48 hours after the treatment, it decreased from 82.66 ± 1.23% at the concentration of 2 μg/ml to 33.91 ± 1.92% at the concentration of 32 μg/ml (P<0.001, Fig.2). In addition, 24 hours after treatment with 4-OH-Ile, viability percentage reached 19.25 ± 5.46% at the concentration of 16 μg/ml and 87.66 ± 1.61% at the concentration of 1μg/ml, and 48 hours after the treatment, it changed from 85.41 ± 3.11% at the concentration of 1 μg/ml to 16.75 ± 2.05% at the concentration of 16 μg/ml (P.0.001, Fig.2). Furthermore, 24 hours after treatment with orlistat, viability percentage reached 34.16 ± 1.69% at the concentration of 48 μg/ ml to 87.57 ± 1.61% at the concentration of 3 μg/ml, and 48 hours after treatment, it was 33.83 ± 1.64% at the concentration of 48 μg/ml and 82.91 ± 1.72% at the concentration of 3 μg/ml (P<0.001, Fig.2). Results also indicated that concentrations <50 μg/ml of HEFS, ≤6.21 μg/ml of diosgenin, ≤1.37 μg/ml of 4-OH-Ile and ≤4.64 μg/ml of orlistat had the minimum inhibitory effect on SW480 cell viability after 24 hours or 48 hours of treatment. Therefore, for the future experiments, the IC20 was used and thus, at this concentration, nearly 80% of the cells had survival potential. In Figure 3, the negative control group, the concentration of IC20 and the concentration of IC50 of SW480 cells, are shown.

### HEFS, diosgenin, 4-OH-Ile and orlistat downregulated the expression of *ACC* and *FAS* genes in SW480 cells

**Fig.4:**
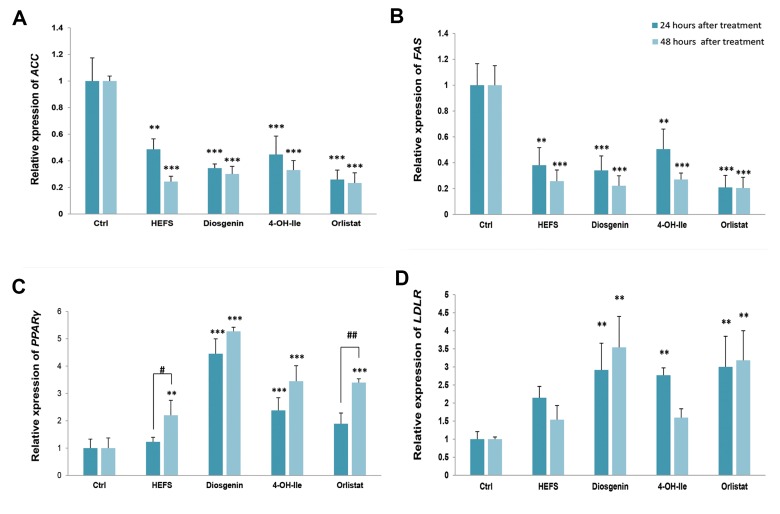
The effects of HFSE, diosgenin, 4-OH-Ile, and orlistat on genes expression in SW480 cells in 24 and 48 hours, respectively. **; P<0.01 and ***; P<0.001 show significant differences compared with the untreated control. #; P<0.05 and ##; P<0.01, and show significant differences between 24 and 48 hours in the indicated groups. **A.** *ACC* (P<0.001, P<0.001), **B.** *FAS* (P<0.001, P<0.001), **C.** *PPARγ* (P<0.001, P<0.001), and **D.** *LDLR* (P=0.005, P=0.001).

Our RT-PCR results showed that 24 and 48 hours treatment with IC20 concentration of HEFS, diosgenin, 4-OH-Ile, and orlistat significantly downregulated the mRNA level of genes involved in lipid metabolism, including *ACC* (0.48-0.34, 0.44-and 0.25, fold decrease- respectively in 24 hours P<0.001) and (0.24-0.30, 0.33-and 0.23, fold decrease-respectively in 48 hours P<0.001) compared to the negative control. After orlistat, the most marked reduction in 24 hours was related to diosgenin (P<0.001) and HEFS (P<0.001) in 48 hours (Fig.4A).

IC20 concentration of the compounds significantly downregulated the expression of FAS (0.38-0.34, 0.50-and 0.20, fold decrease-respectively in 24 hours P<0.001) and (0.25-0.22, 0.27-and 0.20, fold decrease-respectively in 48 hours P<0.001) compared to the negative control. After orlistat, the most marked reduction in 24 and 48 hours was related to diosgenin (P<0.001) (Fig.4B).

### HEFS, diosgenin, 4-OH-Ile and orlistat up-regulated the expression of *PPARγ* and *LDLR *genes in SW480 cells

We also cultured SW480 cells in the presence of the compounds of the expression of *PPARγ* (1.23-4.45, 2.37-and 1.89, fold decrease-respectively for HEFS, diosgenin-4, OH, Ile and orlistat in 24 hours P<0.001) and (2.19-5.27, 3.44, and 3.39-fold decrease, respectively for HEFS-diosgenin, 4, OH, Ile and orlistat in 48 hours P<0.001). These results showed that, diosgenin (P<0.001 and P<0.001 for 24 and 48 hours, respectively), 4-OH-Ile (P=0.035 and P=0.022 for 24 and 48 hours, respectively) and orlistat (P=0.028) in 48 hours significantly reduced the expression of *PPARγ* gene compared to the negative control. Also, there was a significant difference between diosgenin and 4-OH-Ile groups (P=0.001 and P=0.002 for 24 and 48 hours, respectively); diosgenin and orlistat groups (P<0.001, and P=0.001 for 24 and 48 hours, respectively). The independent t test results indicated a significant increase in *PPARγ* gene expression after 48 hours of treatment with HFSE and orlistat compared to *PPARγ* expression in 24 hours (P=0.043) and (P=0.003) respectively for HFSE and orlistat). Overall, among the four compounds used in this study the greatest reduction was related to diosgenin in 24 and 48 hours (P<0.001, Fig.4C).

Also, a significant up-regulation was observed in the expression of *LDLR* (2.14-2.91, 2.76-and 3, fold decrease-respectively in 24 hours P=0.005) and (1.53-3.54, 1.59-and 3.31, fold decrease-respectively in 48 hours P=0.001) genes compared to the negative control. 48-hour results showed significant differences between HFSE and diosgenin groups (P=0.011); HFSE and orlistat groups (P=0.034); diosgenin and 4-OH-Ile groups (P=0.013); and 4-OH-Ile and orlistat groups (P=0.042). The results showed that among the treated groups, the most marked reduction in 24 hours was related to orlistat (P=0.004), while the greatest diminution was related to diosgenin in 48 hours (P=0.001, Fig.4D).

## Discussion

Excessive fat accumulation which is most often due to overeating, leads to obesity and overweight (20). Obesity and overweight, as major health problems, affect all age groups, especially in developing countries (21). Serious social and clinical burdens are imposed by obesity, as reported by researchers. The association between obesity and metabolic syndrome including insulin resistance, type 2 diabetes, heart disease, dyslipidaemia, hypertension and certain types of cancer varying from breast, colon to prostate, is well defined (13). A large body of evidence showed that colon cancers affect obese people more than those with normal weight (22).

Although the initial step for the obesity therapy is lifestyle modification, several synthetic drugs, including orlistat and sibutramine, were designed for obesity, but the safety and efficacy of these drugs are yet to be established. Some medicinal plants were also examined for controlling obesity (23).

Fenugreek, as a medicinal plant, has long been consumed for treatment of metabolic diseases (11). Investigations suggested that the ethanolic extract of fenugreek seeds was able to significantly reduce the plasma level of cholesterol and attenuate the concentrations of liver cholesterol in hypercholesterolemic rats (24). Recent studies reported that fenugreek can be used as a functional supplement for regulation of glucose and lipid profile. Human and animal studies found that fenugreek seeds are rich in fiber, which gives the feeling of satiety and reduces food intake (25). The beneficial effects of fenugreek seeds on the reduction of total cholesterol, TG and LDL-cholesterol levels and hepatic lipid concentrations, were indicated. These effects are due to saponins and diosgenin which are present in fenugreek seeds (26).

It is believed that if the lipid levels, especially TG and LDL-cholesterol are controlled, the risk of several diseases such as type 2 diabetes, metabolic syndrome, insulin resistance, high blood pressure, dyslipidemia, infertility, cardiovascular disease and others, is significantly reduced.

Our findings for the first time, show that HEFS, diosgenin and 4-OH-Ile significantly downregulate *ACC* and *FAS*, while significant up-regulation of *PPARγ* and *LDLR* genes in SW480 cells was similar to changes induced by orlistat following 24 and 48 hours of treatment. Since there is so far no study on the effect of HEFS, diosgenin, 4-OHIle, and orlistat in SW480 cells, here, we refer to similar studies accomplished in other cell lines and animals.

*ACC* is the downstream target of *AMPK* and has been described as a key enzyme in fatty acid biosynthesis where it catalyzes the carboxylation of acetyl-CoA to malonyl- CoA. In the present study, HEFS, diosgenin and 4-OHIle, all decreased the expression of *ACC* gene. Based on the Pyra et al. (27) study, it can possibly be suggested that HEFS and its two bioactive compounds can lead to phosphorylation of *ACC* through phosphorylation of AMPK. Moreover, by reducing the mRNA expression level of *ACC* gene via further phosphorylation, the activity of *ACC* is inhibited and thereby declines the available substrate for *FAS* and, accordingly, de novo fatty acid synthesis. Also, as a result of reducing the content of malonyl-CoA, carnitine palmitoyltransferase I (<italic>CPT- 1</italic>) enzyme, which is the key enzyme in the oxidation of fatty acids, is activated and the beta-oxidation of fatty acids increases (28). These results showed that HEFS and its two bioactive compounds acted in a time-dependent manner, similar to orlistat, and reduced the expression of *ACC* gene. The greatest reduction was related to HEFS in 48 hours. Therefore, it can be said that HEFS probably exerts its hypolipidemic effects via its two bioactive compounds.

One crucial anabolic enzyme required for de novo synthesis of fatty acids is FAS for which, nicotinamide adenine dinucleotide phosphate (*NADPH*) is a cofactor. The present study demonstrated that FAS, as a wellknown and important lipogenic enzyme is downregulated in HEFS, 4-OH-Ile, and diosgenin-treated SW480 cells. It was reported that reduced expression of *FAS* inhibited *de novo* synthesis of fatty acids (29). One study reported that diosgenin reduced the abnormal changes in lipid profile including total cholesterol, triglyceride, and LDL-C. Also, the expressions of *SREBP-1* and its target genes, including *FAS*, (<italic>SCD-1</italic>), and *ACC* were inhibited by diosgenin in rats (30). These results are consistent with our study results. So, it can be suggested that probably, HEFS by its diosgenin content, decreases the expression of *ACC* and *FAS* genes via modulation of *SREBP-1C*.

Our findings demonstrated that HEFS, diosgenin and 4-OH-Ile significantly up-regulated the expression of *PPARγ* gene compared to orlistat. *PPARγ* is a member of the nuclear hormone receptor superfamily that regulates gene expression by binding to DNA and plays an important role in lipid homeostasis. It is highly expressed in white and brown adipose tissues, however, it is also expressed by the colon, liver, and muscle (31). Unsaturated fatty acids and their derivatives are endogenous ligands for PPARs. After binding to ligand, PPARs after heterodimerization with retinoic X receptor (*RXR*), bind to *PPAR* response elements (*PPREs*) in the regulatory region of several target genes (32). *PPARγ* is a positive regulator of adiponectin (*ADN*) gene expression. *ADN* increases fatty acid oxidation and limits the endogenous synthesis of lipids by reducing the circulating level of free fatty acids (33). So, HEFS and its two bioactive compounds act like PPAR ligands and upregulate the expression of *PPARγ* hence enhancing the level of ADN by therapeutic agents might be helpful in the treatment of obesity and overweight. A study determined that three phytochemicals namely, kaempferol, curcumin and puerarin moderate the expression and activity of organic anion/cation transporter 2 (*OCTN_2_*) by activation of the *PPARg/RXRa* pathway in SW480 cell line. *OCTN_2_* is a member of the solute carrier transporters, which are expressed in human tissues including the kidney, brain, heart, small intestine and colon and it plays a role in the transfer of many endogenous substrates, including carnitine (34). Carnitine is required for mitochondrial â-oxidation of fatty acids (35). Furthermore, we hypothesized that an increase in *PPARγ* leads to an increase in *OCTN_2_* and subsequently an enhancement in carnitine and fatty acids beta-oxidation.

A previous study reported that diosgenin inhibited the differentiation of adipocytes in 3T3-L1 cells by suppressing the expression of *PPARγ* gene and its target genes. In fact, diosgenin increases the expression of estrogen receptor β (*ERβ*), after which ERβ forms a heterodimer with RXRα, and RXRα is separated from PPARγ in the PPARγ/ RXRa complex, which reduces PPARγ transcriptional activity. Thus, the expression of *PPARγ* in adipocytes was significantly affected by diosgenin, but in our study, the expression of *PPARγ* in SW480 cells was significantly increased because *PPARγ* in the colon plays a different role from adipose tissue. Its mechanism may be mediated via the liver X receptor (*LXR*). *LXRs* belong to the nuclear hormone receptor superfamily. Studies showed that *LXRα* with *RXR* forms a heterodimer complex, and then, this complex attaches to the cysteine elements found in the promoter of *SREBP-1C* gene and activates transcription of this gene, so, it regulates lip o genesis. Additionally, like *LXRs*, activated *PPARs* also he t erodimerize with the *RXR* and alter the transcriptio n of target genes. Thus, overexpression of *PPARγ* in S W 480 cells under the influence of HEFS, diosgenin, 4-OH-Ile, and orlistat competes with *LXRα/RXR* heterodimer ization, resulting in a reduction in the transcripti o n of the *SREBP- 1C* and its target genes including *ACC* and *FAS* (36). Though considerable attention has been paid to the antiinflammatory (37) and anti-carcinogenic role of *PPARγ* in the colon (38), *PPARγ* is believed to act as a basic lipid sensor controlling the expressio n of genes involved in carbohydrate and lipid metabolism, resulting in increased expression of lipoprotein lipase (*LPL*) and decreased expression of apolipoprotein *(apo)* *C-III*, both key-players in plasma TG metabolism. Moreover, as a downstream target gene of *PPARγ*, *CD36* is known as a mediator in long chain fatty acid (*LCFA*) upt a ke. Consequently, TG accumulation via *LPL* and rise in beta-oxidation of fatty acids by CD36 in the bowel, can be inhibited by an increase in *PPARy* (39).

We illustrated that HEFS, diosgenin, and 4-OH-Ile treatment significantly increased the expression of the genes coding *LDL* receptor (*LDLR*) in SW480 cells. Reduced cell surface *LDLR* expression leads to an increase in LDL in the circulation. Also, impairment of the *LDLR* activity results in the accumulation of LDL particles in the flow, inducing atherosclerosis development (40). Thus, HEFS and its two bioactive compounds may be beneficial because of their protective effect on obesity.

The overall results of this study showed that among the groups treated with HEFS, diosgenin, and 4-OH-Ile, the most significant effect was related to diosgenin. Thus, most of the hypolipidemic effects of HEFS are probably caused by diosgenin. Our study showed results similar to those of studies done in the liver cells; so, there is similarity in the effects of fenugreek compounds in the liver and colon cells with respect to fatty acid metabolism. However, HEFS and its derivatives should be further investigated for their effects on dyslipidemia and its complications.

## Conclusion

Overall, these results showed significant downregulation of *ACC* and *FAS* alongside upregulation of *PPARγ* and *LDLR* genes at mRNA level in SW480 cell lines treated with HEFS, diosgenin, and 4-OH-Ile. These results present evidence for the hypolipidemic activity of HEFS and its two active substances similar to orlistat. Therefore, according to our findings, they may be suggested as a useful natural remedy for controlling obesity and overweight.

For future studies, studying the effects of the four substances used in this study in other cell lines, in particular the fat cell line (3T3L-1), evaluation of other genes involved in fat metabolism, using other techniques such as western blot and immunohistochemistry to evaluate the protein level of these genes, are recommended by the authors.
